# Effects of Endocrine Metabolic Factors on Hemocyte Parameters, Tumor Markers, and Blood Electrolytes in Patients with Hyperglycemia

**DOI:** 10.1155/2023/8905218

**Published:** 2023-04-11

**Authors:** Lin Qin, Yan-Jun Chen, Ting-Hua Wang, Rong-Ping Zhang

**Affiliations:** ^1^Department of Endocrinology, First Affiliated Hospital of Kunming Medical University, Kunming 650032, Yunnan, China; ^2^Institute of Neuroscience, Kunming Medical University, Kunming 650032, Yunnan, China; ^3^College of Chinese Materia Medica and Yunnan Key Laboratory of Southern Medicine Utilization, Yunnan University of Chinese Medicine, Kunming 650500, Yunnan, China

## Abstract

**Objective:**

This study was designed to investigate the effect of endocrine metabolic factors on hemocyte parameters, tumor markers, and blood electrolytes in patients with hyperglycemia.

**Methods:**

In this study, 1791 patients with hyperglycemia were recruited and grouped according to different testing indexes, and their medical records and laboratory indexes were recorded and analyzed.

**Results:**

In adult patients with hyperglycemia, we found that high-density lipoprotein cholesterol (HDL-C) was negatively correlated with white blood cell (WBC) and could exert an effect on WBC; triglyceride (TG) level was positively associated with lymphocyte (LYM#); age, TG, and P affected the level of LYM#; and uric acid (UA) level was positively related to eosinophil (EO#). Besides, age was positively correlated with red blood cell distribution width-coefficient of variation (RDW-CV) level; fasting blood glucose (FBG) and serum phosphorus (P) were negatively correlated with RDW-CV level; and age, creatinine (Cre), FBG, HDL-C, and P were influencing factors of RDW-CV level in adult hyperglycemic patients. HDL-C was negatively correlated with fibrinogen (Fib) level, and age, HDL-C, serum kalium (K), serum sodium (Na), and body mass index (BMI) were the influencing factors of Fib levels. TG was positively associated with neuron-specific enolase (NSE) level and affected the NSE level. Serum magnesium (Mg) was negatively related to carcinoembryonic antigen (CEA) level, and sex, age, FBG, Mg, and BMI could have an effect on CEA level. As well, age and FBG were positively associated with carbohydrate antigen 50 (CA50) levels, UA was negatively correlated with CA50 levels, and age, aspartate aminotransferase (AST), UA, and FBG were the influencing factors of CA50 levels. FBG was negatively related to Mg levels; K, serum zinc (Zn), and fasting C-peptide (C-P) were positively correlated with Mg levels; and FBG, K, Zn, and C-P had an effect on Mg levels.

**Conclusion:**

Endocrine metabolic factors are closely related to hemocyte parameters, tumor markers, and blood electrolytes in patients with hyperglycemia.

## 1. Introduction

In recent years, the incidence of hyperglycemia in China has been increasing year by year, and diabetes and its complications have become a serious threat to the health of patients. Endocrine metabolic factors are closely related to blood cell parameters, tumor markers, and blood electrolytes. It has been reported that the physiological changes occurring in the hyperglycemic state may lead to adverse outcomes [1]. Elevated blood glucose levels impair neutrophil function and lead to the overproduction of reactive oxygen species, free fatty acids, and inflammatory mediators [2]. These pathophysiological changes directly lead to cellular damage and vascular and immune dysfunction [3, 4]. Therefore, this study is conducted to investigate whether blood cell parameters, tumor markers, and blood electrolytes of patients with hyperglycemia are affected by endocrine metabolic indicators to provide a reference for the clinical diagnosis and treatment.

## 2. Methods

All procedures were approved by the Ethics Committee of First Affiliated Hospital of Kunming Medical University (approval no. kmmu2021426), and the application for the exemption from informed consent was approved. The study was conducted in accordance with the principles of the Declaration of Helsinki.

### 2.1. Patients

Patients with hyperglycemia hospitalized in the Department of Endocrinology, the First Affiliated Hospital of Kunming Medical University, from January 2016 to September 2020 were recruited in the study. Inclusion criteria were that patients met the diagnostic criteria of hyperglycemia, including prediabetic and diabetic patients who met the 1999 World Health Organization diagnostic criteria. Exclusion criteria were as follows: (1) patients who did not meet the diagnosis of diabetes mellitus or impaired glucose regulation; (2) patients who were under 18 years of age; (3) patients with incomplete clinical data; and (4) patients with diseases that affect the white blood cell (WBC) count and lymphocyte count, such as leukemia.

### 2.2. Grouping

Patients were grouped according to the different indicators analyzed as follows:

1. 1791 patients were assigned into the WBC ≤ 4 × 10^9^/L group (*n* = 90) and WBC > 4 × 10^9^/L group (*n* = 1701).
2. 1604 patients were assigned into absolute lymphocyte count (LYM#) ≤ 1.10 × 10^9^/L group (*n* = 63) and LYM#>1.10 × 10^9^/L group (*n* = 1541).
3. 1766 patients were assigned into absolute eosinophil count (EO#) ≤ 0.14 × 10^9^/L group (*n* = 924) and EO#>0.14 × 10^9^/L group (*n* = 842).
4. 1765 patients were assigned into red blood cell distribution width − coefficient of variation (RDW − CV) ≤ 12.90 g/L group (*n* = 903) and RDW − CV > 12.90 g/L group (*n* = 862).
5. 1610 patients were assigned into the fibrinogen (Fib) ≤ 4 g/L group (*n* = 1441) and Fib > 4 g/L group (*n* = 169).
6. 773 patients were assigned into the neuron − specific enolase (NSE) ≤ 16.3 ng/mL group (*n* = 753) and NSE > 16.3 ng/mL group (*n* = 20).
7. 770 patients were assigned into the carcinoembryonic antigen (CEA) ≤ 5.0 ng/mL group (*n* = 668) and the CEA > 5.0 ng/mL group (*n* = 102).
8. 648 patients were assigned into the carbohydrate antigen 50 (CA50) ≤ 7.20 U/mL group (*n* = 324) and CA50 > 7.20 U/mL group (*n* = 324).
9. 995 patients were assigned into the serum magnesium (Mg) ≤ 0.75 mmol/L group (*n* = 141) and Mg > 0.75 mmol/L group (*n* = 854).

### 2.3. Testing Indicators

The patients' sex, age, body mass index (BMI), and laboratory indexes were recorded and analyzed, and the peripheral venous blood sample of patients was collected at 12 hours after fasting for routine blood analysis and blood biochemical analysis. The testing indexes included aspartate aminotransferase (AST), alanine aminotransferase (ALT), creatinine (Cre), uric acid (UA), fasting blood glucose (FBG), total cholesterol (TC), free cholesterol (F-CHOL), triglycerides (TG), high-density lipoprotein cholesterol (HDL-C), low-density lipoprotein cholesterol (LDL-C), serum kalium (K), serum sodium (Na), serum chloride (Cl), serum calcium (Ca), Mg, serum phosphorus (P), serum zinc (Zn), NSE, CEA, CA50, and fasting C-peptide (C-P).

### 2.4. Statistical Analysis

All data were imported into SPSS 25.0 for statistical analysis. Quantitative data was performed with an independent sample *t*-test or Mann–Whitney *U* test, and *χ*^2^ test was used for the comparison of qualitative data. The correlation between variables was analyzed by the Pearson correlation or the Spearman correlation analysis, and the regression relation was analyzed by logistic regression analysis. ^∗^*P* < 0.05 was considered a statistically significant difference.

## 3. Results

### 3.1. Endocrine Metabolic Indicators and Blood Cell Parameters

#### 3.1.1. Influencing Factors of WBC

**Figure 1:**
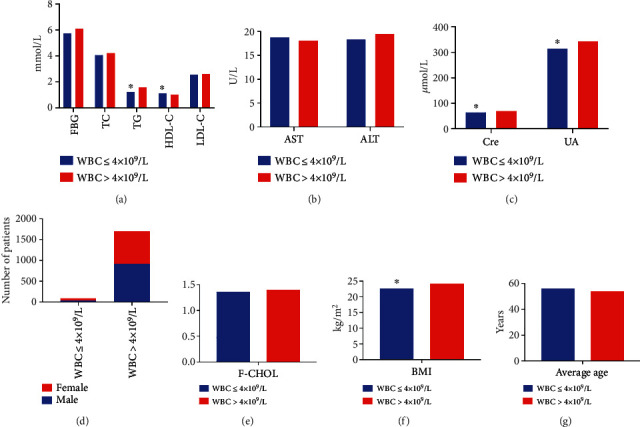
Comparison of different indicators between the WBC ≤ 4 × 10^9^/L and WBC > 4 × 10^9^/L groups. (a) Comparison of levels of FBG, TC, TG, HDL-C, and LDL-C between the two groups. The levels of TG and HDL-C in the WBC ≤ 4 × 10^9^/L group were significantly lower than that in WBC > 4 × 10^9^/L group. (b) Comparison of AST and ALT levels between the groups. (c) The levels of Cre and UA in the WBC ≤ 4 × 10^9^/L group were significantly lower than that in the WBC > 4 × 10^9^/L group. (d) Comparison of the number of male and female patients between the groups. (e) Comparison of levels of F-CHOL between the groups. (f) The BMI of patients in the WBC ≤ 4 × 10^9^/L group was significantly lower than that in the WBC > 4 × 10^9^/L group. (g) Comparison of the average age of patients between the two groups.

The levels of Cre, UA, TG, and BMI in the WBC > 4 × 10^9^/L group were significantly higher, and the HDL-C level was lower in comparison with the WBC < 4 × 10^9^/L group (*P* < 0.05), and no difference was observed in sex, age, AST, ALT, FBG, TC, F-CHOL, and LDL-C between the groups (*P* > 0.05, Table 1 and Figure 1). Correlation analysis showed that the WBC was associated with HDL-C (*r* = −0.160), age (*r* = −0.193), ALT (*r* = 0.073), Cre (*r* = 0.091), UA (*r* = 0.150), TG (*r* = 0.194), and BMI (*r* = 0.195), and the bias correlation analysis showed a significant negative correlation between WBC and HDL-C (*r* = −0.070) (*P* < 0.05). Logistic regression analysis showed that HDL-C (OR = 0.432, 95%CI = 0.222 − 0.842) and BMI (OR = 1.128, 95%CI = 1.058 − 1.202) had a regression relation with whether the WBC ≤ 4 × 10^9^/L (*P* < 0.05).

#### 3.1.2. Factors Affecting the Reduction of LYM#

**Figure 2:**
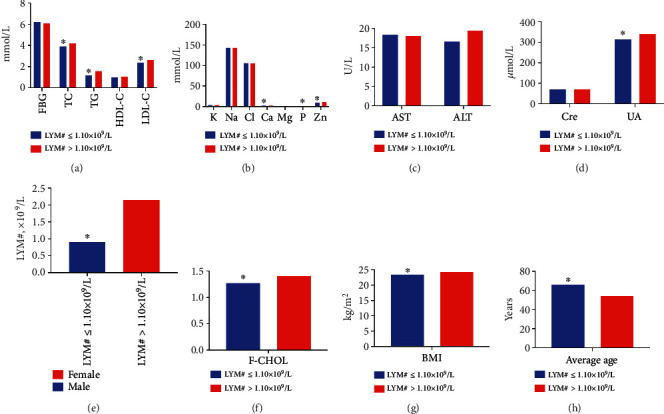
Comparison of different indicators between the LYM#≤1.10 × 10^9^/L and LYM#>1.10 × 10^9^/L groups. (a) Comparison of levels of FBG, TC, TG, HDL-C, and LDL-C between the two groups. The levels of TC, TG, and LDL-C in LYM#≤1.10 × 10^9^/L group were significantly lower than that in LYM#>1.10 × 10^9^/L group. (b) Comparison of levels of K, Na, Cl, Ca, Mg, P, and Zn in the blood between the two groups. The levels of blood Ca, P, and Zn were significantly lower in the LYM#≤1.10 × 10^9^/L group than that in the LYM#>1.10 × 10^9^/L group. (c) Comparison of AST and ALT levels between the groups. (d) Comparison of Cre and UA levels between the groups. The levels of UA in LYM#≤1.10 × 10^9^/L group were significantly lower than that in LYM#>1.10 × 10^9^/L group. (e) The LYM# in LYM#≤1.10 × 10^9^/L group was significantly lower than that in LYM#>1.10 × 10^9^/L group. (f) The F-CHOL of patients in LYM#≤1.10 × 10^9^/L group was significantly lower than that in LYM#>1.10 × 10^9^/L group. (g) The BMI of patients in the LYM#≤1.10 × 10^9^/L group was significantly lower than that in LYM#>1.10 × 10^9^/L group. (h) The average age of patients in the LYM#≤1.10 × 10^9^/L group was significantly higher than in LYM#>1.10 × 10^9^/L group.

It was showed that LYM#, UA, TC, F-CHOL, TG, LDL-C, Ca, P, Zn, and BMI in the LYM#>1.10 × 10^9^/L group were markedly higher than that in LYM#≤1.10 × 10^9^/L, while the average age of patients was lower (*P* < 0.05), and there were no differences in AST, ALT, Cre, FBG, HDL-C, K, Na, Cl, and Mg between the groups (*P* > 0.05, Table 2 and Figure 2). Spearman analysis indicated that age (*r* = −0.266), ALT (*r* = −0.113), Cre (*r* = −0.087), UA (*r* = −0.075), TC (*r* = −0.132), F-CHOL (*r* = −0.114), TG (*r* = −0.168), HDL-C (*r* = −0.065), LDL-C (*r* = −0.136), Cl (*r* = −0.060), Ca (*r* = −0.118), P (*r* = −0.229), Zn (*r* = −0.084), and BMI (*r* = −0.133) were associated with LYM#, and the bias correlation analysis showed a significant positive correlation between TG (*r* = 0.095, *P* = 0.001) and P (*r* = 0.144) levels and LYM# (*P* < 0.05). Logistic regression analysis showed that age (OR = 0.966, 95%CI = 0.946 − 0.987), TG (OR = 1.804, 95%CI = 1.007 − 3.231), and P (OR = 74.349, 95%CI = 18.151 − 304.543) had a regression relation with whether LYM#≤1.10 × 10^9^/L (*P* < 0.05).

#### 3.1.3. Influencing Factors of EO#

**Figure 3:**
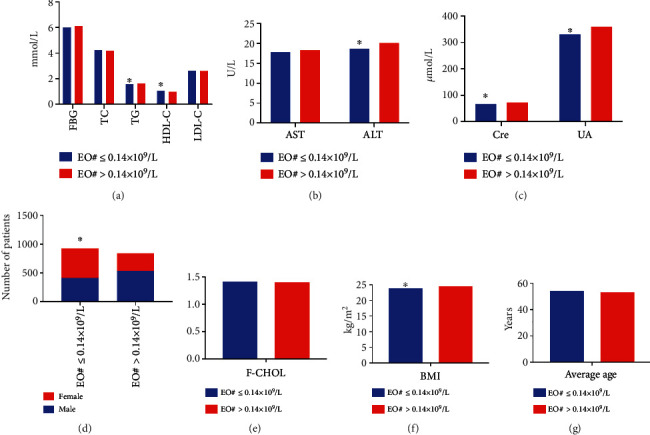
Comparison of different indicators between the EO#≤0.14 × 10^9^/L and EO#>0.14 × 10^9^/L groups. (a) Comparison of levels of FBG, TC, TG, HDL-C, and LDL-C between the two groups. Compared with the EO#>0.14 × 10^9^/L group, the levels of TG were significantly lower and HDL-C was higher in EO#≤0.14 × 10^9^/L group. (b) Comparison of AST and ALT levels between the groups. The levels of ALT were significantly lower in EO#≤0.14 × 10^9^/L group than in the EO#>0.14 × 10^9^/L group.(c) The levels of Cre and UA in EO#≤0.14 × 10^9^/L group were significantly lower than that in EO#>0.14 × 10^9^/L group. (d) Comparison of the number of male and female patients between the groups. (e) Comparison of levels of F-CHOL between the groups. (f) The BMI of patients in WBC ≤ 4 × 10^9^/L group was significantly lower than that in WBC > 4 × 10^9^/L group. (g) Comparison of the average age of patients between the two groups.

As presented in Table 3, the ALT, Cre, UA, TG, and BMI in the EO#>0.14 × 10^9^/L group were obviously higher compared with the EO#≤0.14 × 10^9^/L group, but the HDL-C levels were decreased in the EO#>0.14 × 10^9^/L group (*P* < 0.05, Figure 3). However, no significant differences were found in the age, AST, FBG, TC, F-CHOL, and LDL-C levels between the groups (*P* > 0.05). Spearman analysis showed that levels of ALT (*r* = 0.056, *P* = 0.019), Cre (*r* = 0.160), UA (*r* = 0.177), TG (*r* = 0.085), HDL-C (*r* = −0.127), and BMI (*r* = 0.104) were correlated with EO#, and the bias correlation analysis exhibited a significant positive correlation between UA levels and EO# (*r* = 0.084, *P* < 0.05). Logistic regression analysis showed that male (OR = 1.870, 95% CI = 1.513 − 2.311), ALT (OR = 1.005, 95%CI = 1.001 − 1.009), UA (OR = 1.001, 95%CI = 1.000 − 1.002), and TG (OR = 1.050, 95%CI = 1.000 − 1.102) were regressed with EO#≤0.14 × 10^9^/L (*P* < 0.05).

#### 3.1.4. Influencing Factors of RDW-CV

**Figure 4:**
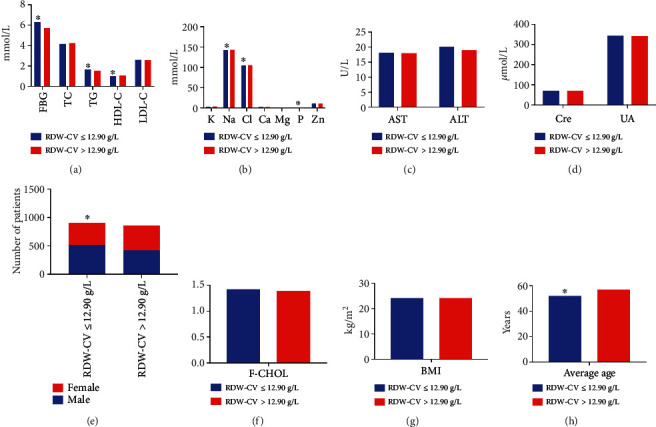
Comparison of different indicators between the RDW − CV ≤ 12.90 g/L and RDW − CV > 12.90 g/L groups. (a) Comparison of levels of FBG, TC, TG, HDL-C, and LDL-C between the two groups. Compared with the RDW − CV > 12.90 g/L group, the levels of FBG and TG were significantly higher but levels of HDL-C were lower in RDW − CV ≤ 12.90 g/L group. (b) Comparison of levels of K, Na, Cl, Ca, Mg, P, and Zn in the blood between the two groups. The levels of blood Na, Cl, and P were significantly lower in the RDW − CV ≤ 12.90 g/L group than that in the RDW − CV > 12.90 g/L group. (c) Comparison of AST and ALT levels between the groups. (d) Comparison of Cre and UA levels between the groups. (e) Comparison of the number of male and female patients between the groups. (f) Comparison of levels of F-CHOL between the groups. (g) Comparison of BMI of patients between the groups. (h) The average age of patients in RDW − CV ≤ 12.90 g/L group was significantly higher than in RDW − CV > 12.90 g/L group.

Compared with the RDW − CV ≤ 12.90 g/L group, the average age, male, HDL-C, Na, and Cl levels were remarkably increased, and the female, FBG, TG, and P levels were significantly decreased in the RDW − CV > 12.90 g/L group of patients with hyperglycemia (*P* < 0.05); no statistically significant difference was observed in the AST, ALT, Cre, UA, TC, F-CHOL, LDL-C, K, Ca, Mg, Zn, and BMI between the groups (Table 4 and Figure 4). Spearman analysis demonstrated that age (*r* = 0.203), ALT (*r* = −0.057), Cre (*r* = 0.050), FBG (*r* = −0.145), TG (*r* = −0.067), HDL-C (*r* = 0.102), Na (*r* = 0.123), Cl (*r* = 0.136), P (*r* = −0.111), and Zn (*r* = −0.055) were associated with RDW-CV levels (*P* < 0.05). Moreover, the bias correlation analysis exhibited a significant positive correlation between age and RDW-CV levels (*r* = 0.140) and a markedly negative correlation between the FBG and serum P levels and RDW-CV levels (*P* < 0.05). Logistic regression analysis showed that male (OR = 0.727, 95%CI = 0.581 − 0.910), age (OR = 1.019, 95%CI = 1.011 − 1.027), Cre (OR = 1.005, 95%CI = 1.001 − 1.009), FBG (OR = 0.949, 95%CI = 0.914 − 0.987), HDL-C (OR = 1.474, 95%CI = 1.036 − 2.009), and P (OR = 0.517, 95%CI = 0.318 − 0.840) levels had an effect on RDW-CV levels (*P* < 0.05).

### 3.2. Effect of Endocrine Metabolic Factors on Fib Levels

**Figure 5:**
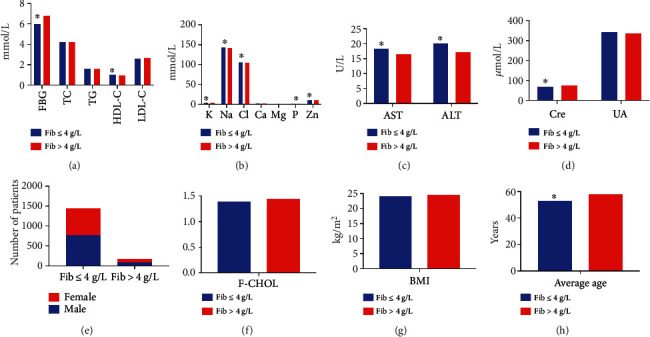
Comparison of different indicators between the Fib ≤ 4 g/L and Fib > 4 g/L groups. (a) Comparison of levels of FBG, TC, TG, HDL-C, and LDL-C between the two groups. Compared with the Fib > 4 g/L group, the levels of FBG were significantly lower but levels of HDL-C were higher in Fib ≤ 4 g/L group. (b) Comparison of levels of K, Na, Cl, Ca, Mg, P, and Zn in the blood between the two groups. Significant differences were observed in K, Na, Cl, P, and Zn between the groups. (c) The levels of AST and ALT in Fib ≤ 4 g/L group were significantly higher than that in Fib > 4 g/L group. (d) Comparison of Cre and UA levels between the groups. The levels of Cre were significantly lower in Fib ≤ 4 g/L group than that in Fib > 4 g/L group. (e) Comparison of the number of male and female patients between the groups. (f) Comparison of levels of F-CHOL between the groups. (g) Comparison of BMI of patients between the groups. (h) The average age of patients in Fib ≤ 4 g/L group was significantly lower than in Fib > 4 g/L group.

The age, Cre, FBG, and serum K levels were significantly higher in the Fib > 4 g/L group, and AST, ALT, HDL-C, Na, Cl, P, and Zn levels were decreased, compared with Fib ≤ 4 g/L group (*P* < 0.05, Table 5 and Figure 5). No statistically significant difference was observed in sex, UA, TC, F-CHOL, TG, LDL-C, Ca, Mg, and BMI between the groups (*P* > 0.05). Spearman analysis suggested that age (*r* = 0.142, *P* = 0.000), ALT (*r* = −0.084), FBG (*r* = 0.064), TC (*r* = 0.107), F-CHOL (*r* = 0.115), HDL-C (*r* = −0.058), LDL-C (*r* = 0.110), K (*r* = 0.171), Na (*r* = −0.108), Cl (*r* = −0.052), Ca (*r* = 0.097), Mg (*r* = 0.053), and BMI (*r* = 0.085) were associated with Fib levels, and the bias correlation analysis presented a markedly negative correlation between HDL-C and Fib levels (*r* = −0.128) (*P* < 0.05). Logistic regression analysis indicated that age (OR = 1.028, 95%CI = 1.015 − 1.042), HDL-C (OR = 0.506, 95%CI = 0.258 − 0.991), K (OR = 1.911, 95%CI = 1.253 − 2.913), Na (OR = 0.859, 95%CI = 0.786 − 0.939), and BMI (OR = 1.055, 95% CI = 1.010 − 1.103) were risk factors of the Fib levels (*P* < 0.05).

### 3.3. Endocrine Metabolic Factors and Tumor Markers

#### 3.3.1. Influencing Factors of NSE Levels

**Figure 6:**
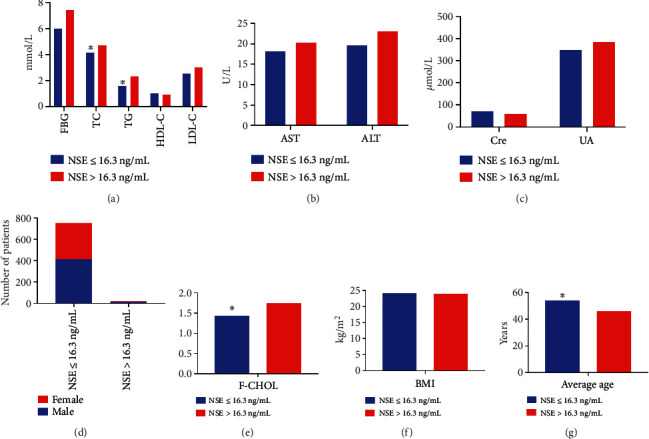
Comparison of different indicators between the NSE ≤ 16.3 ng/mL and NSE > 16.3 ng/mL groups. (a) Comparison of levels of FBG, TC, TG, HDL-C, and LDL-C between the two groups. Compared with the NSE > 16.3 ng/mL group, the levels of TG and TC were significantly lower in NSE ≤ 16.3 ng/mL group. (b) Comparison of AST and ALT levels between the groups. (c) Comparison of Cre and UA levels between the groups. (d) Comparison of the number of male and female patients between the groups. (e) The levels of F-CHOL were significantly lower in NSE ≤ 16.3 ng/mL group. (f) Comparison of BMI of patients between the groups. (g) The average age of patients in NSE ≤ 16.3 ng/mL group was significantly higher than in NSE > 16.3 ng/mL group.

Compared with the NSE ≤ 16.3 ng/mL group, the levels of TC, F-CHOL, and TG were remarkably increased in the NSE > 16.3 ng/mL group, while the age of patients in the NSE > 16.3 ng/mL group was lower (*P* < 0.05); however, there was no difference in sex, AST, ALT, Cre, UA, FBG, HDL-C, LDL-C, and BMI between the groups (*P* > 0.05, Table 6 and Figure 6). Spearman correlation analysis showed that NSE level was associated with levels of TG (*r* = 0.092) and HDL-C (*r* = −0.075) (*P* < 0.05). The bias correlation analysis presented a significantly positive correlation between HDL-C and NSE levels (*r* = 0.078), and there was a regression relation between the TG (OR = 1.259, 95%CI = 1.130 − 1.403) and NSE levels (*P* < 0.05).

#### 3.3.2. Influencing Factors of CEA Levels

**Figure 7:**
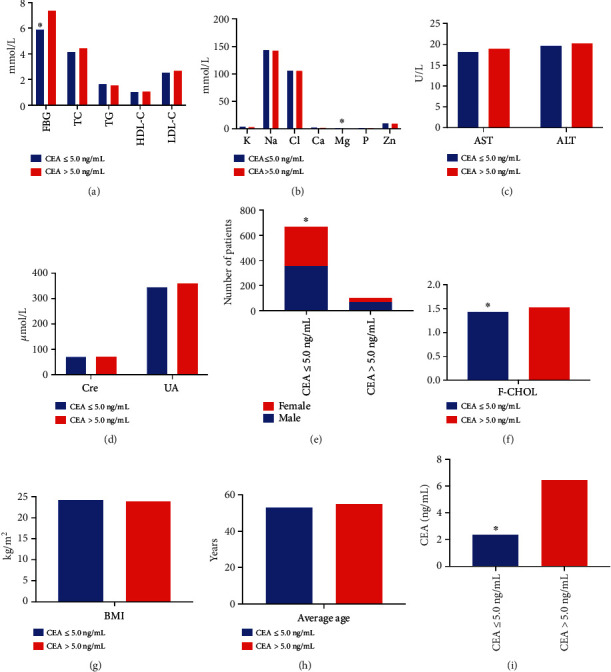
Comparison of different indicators between the CEA ≤ 5.0 ng/mL and CEA > 5.0 ng/mL groups. (a) Comparison of levels of FBG, TC, TG, HDL-C, and LDL-C between the two groups. Compared with the CEA > 5.0 ng/mL group, the levels of FBG were significantly lower in CEA ≤ 5.0 ng/mL group. (b) Comparison of levels of K, Na, Cl, Ca, Mg, P, and Zn in the blood between the two groups. (c) Comparison of AST and ALT levels between the groups. (d) Comparison of Cre and UA levels between the groups. (e) Comparison of the number of male and female patients between the groups. (f) The F-CHOL in CEA ≤ 5.0 ng/mL group was significantly lower than in CEA > 5.0 ng/mL group. (g) Comparison of BMI of patients between the groups. (h) Comparison of average age of patients between the groups. (i) The blood CEA in CEA ≤ 5.0 ng/mL group was significantly lower than in CEA > 5.0 ng/mL group.

As presented in Table 7, the levels of CEA, FBG, and F-CHOL in the CEA > 5.0 ng/mL group were obviously higher compared with the CEA ≤ 5.0 ng/mL group, but the number of male/female and serum Mg levels was decreased in the CEA > 5.0 ng/mL group (*P* < 0.05, Figure 7). No statistically significant differences were observed in the age, AST, ALT, Cre, UA, TC, TG, HDL-C, LDL-C, K, Na, Cl, Ca, P, Zn, and BMI between the groups (*P* > 0.05, Figure 7). Spearman analysis showed that age (*r* = 0.157), FBG (*r* = 0.254), F-CHOL (*r* = 0.110), K (*r* = −0.110), Ca (*r* = −0.084), Mg (*r* = −0.141), P (*r* = −0.101), and BMI (*r* = −0.074) were associated with CEA levels, and the bias correlation analysis presented a negative correlation between Mg and CEA levels (*r* = −0.091) (*P* < 0.05). Logistic regression analysis showed that sex (OR = 2.100, 95%CI = 1.307 − 3.374), age (OR = 1.028, 95%CI = 1.008 − 1.049), FBG (OR = 1.163, 95%CI = 1.082 − 1.251), Mg (OR = 0.039, 95%CI = 0.003 − 0.569), and BMI (OR = 0.925, 95%CI = 0.865 − 0.988) had an effect on CEA levels (*P* < 0.05).

#### 3.3.3. Influencing Factors of CA50 Levels

**Figure 8:**
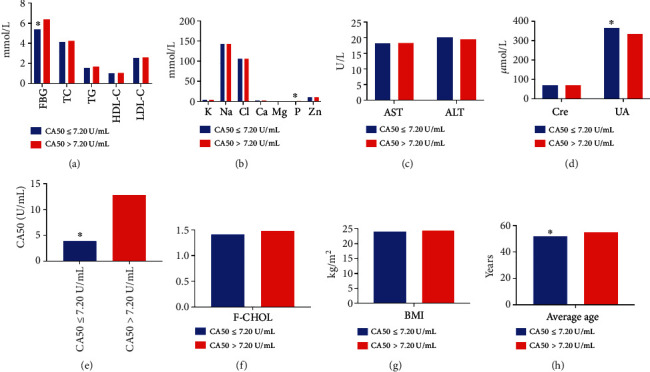
Comparison of different indicators between the CA50 ≤ 7.2 U/mL and CA50 > 7.2 U/mL groups. (a) Comparison of levels of FBG, TC, TG, HDL-C, and LDL-C between the two groups. Compared with the CA50 > 7.2 U/mL group, the levels of FBG were significantly lower in CA50 ≤ 7.2 U/mL group. (b) Comparison of levels of K, Na, Cl, Ca, Mg, P, and Zn in the blood between the two groups. (c) Comparison of AST and ALT levels between the groups. (d) Comparison of Cre and UA levels between the groups. The levels of UA in CA50 ≤ 7.2 U/mL group were significantly higher than that in CA50 > 7.2 U/mL group. (e) The CA50 in CA50 ≤ 7.2 U/mL group was significantly lower than that in CA50 > 7.2 U/mL group. (f) Comparison of F-CHOL between the groups. (g) Comparison of BMI of patients between the groups. (h) The average age of patients in CA50 ≤ 7.2 U/mL group was significantly lower than in CA50 > 7.2 U/mL group.

It was shown that the average age, CA50, and FBG levels were obviously higher in the CA50 > 7.20 U/mL group, while the UA and serum P levels were lower in comparison with the CA50 ≤ 7.20 U/mL group (*P* < 0.05); no statistically significant differences were observed in the levels of AST, ALT, Cre, TC, F-CHOL, TG, HDL-C, LDL-C, K, Na, Cl, Ca, Mg, Zn, and BMI between the groups (*P* > 0.05, Table 8 and Figure 8). Spearman analysis showed that CA50 levels were associated with age (*r* = 0.168), AST (*r* = 0.085), UA (*r* = −0.128), FBG (*r* = 0.221), and Na (*r* = −0.085) (*P* < 0.05). Moreover, the bias correlation analysis exhibited a significant positive correlation between age (*r* = 0.150), FBG (*r* = 0.188), and CA50 levels and a markedly negative correlation between the UA and CA50 levels (*r* = −0.103) (*P* < 0.05). Logistic regression analysis showed that age (OR = 1.031, 95%CI = 1.016 − 1.046), AST (OR = 1.022, 95%CI = 1.008 − 1.036), UA (OR = 0.997, 95%CI = 0.996 − 0.999), and FBG (OR = 1.186, 95%CI = 1.110 − 1.267) could have an effect on blood CA50 levels (*P* < 0.05).

### 3.4. Endocrine Metabolic Factors and Mg Levels

**Figure 9:**
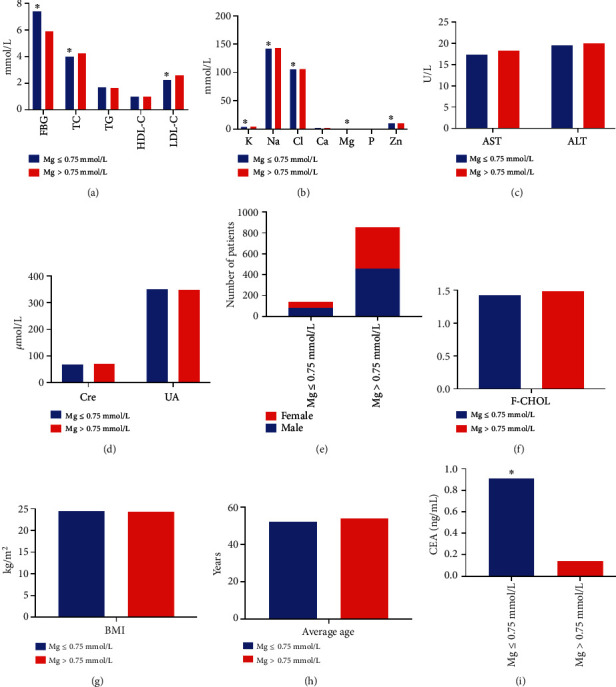
Comparison of different indicators between the Mg ≤ 0.75 mmol/L and Mg > 0.75 mmol/L groups. (a) Comparison of levels of FBG, TC, TG, HDL-C, and LDL-C between the two groups. Compared with the Mg > 0.75 mmol/L group, the levels of FBG and TC were significantly higher but LDL-C was lower in Mg ≤ 0.75 mmol/L group. (b) Comparison of levels of K, Na, Cl, Ca, Mg, P, and Zn in the blood between the two groups. (c) Comparison of AST and ALT levels between the groups. (d) Comparison of Cre and UA levels between the groups. (e) Comparison of the number of male and female patients between the groups. (f) Comparison of F-CHOL between the groups. (g) Comparison of BMI of patients between the groups. (h) Comparison of the average age of patients between the groups. (i) The blood CEA in Mg ≤ 0.75 mmol/L group was significantly higher than in Mg > 0.75 mmol/L group.

Compared with the Mg ≤ 0.75 mmol/L group, the FBG levels were decreased and the TC, LDL-C, K, Na, Cl, Mg, Zn, and C-P levels were increased in the Mg > 0.75 mmol/L group (*P* < 0.05). No difference was found in the sex, age, AST, ALT, Cre, UA, F-CHOL, TG, HDL-C, Ca, P, and BMI between the groups (*P* > 0.05, Table 9 and Figure 9). Spearman analysis showed that age (*r* = 0.088), AST (*r* = 0.071), Cre (*r* = 0.129), FBG (*r* = −0.226), TC (*r* = 0.089), HDL-C (*r* = 0.078), LDL-C (*r* = 0.111), K (*r* = 0.201), Na (*r* = 0.153), Cl (*r* = 0.135), Zn (*r* = 0.122), and C-P levels (*r* = 0.147) were associated with Mg levels (*P* < 0.05). In addition, the bias correlation analysis exhibited a significant positive correlation between C-P (*r* = 0.195), K (*r* = 0.187), Zn (*r* = 0.146), and CA50 levels and a negative correlation between the FBG and CA50 levels (*r* = −0.203) (*P* < 0.05). Logistic regression analysis showed that FBG (OR = 0.890, 95%CI = 0.833 − 0.951), K (OR = 2.624, 95%CI = 1.589 − 4.335), Zn (OR = 1.140, 95%CI = 1.037 − 1.252), and C-P (OR = 1.383, 95%CI = 1.076 − 1.776) were the influencing factors of Mg levels (*P* < 0.05).

## 4. Discussion

In this study, we investigated the correlation of endocrine metabolic indicators with blood cell parameters, tumor markers, and blood electrocytes in adult patients with hyperglycemia.

Our findings demonstrated that HDL-C levels were higher in the WBC ≤ 4 × 10^9^/L group than in the WBC > 4 × 10^9^/L group of adult patients with hyperglycemia, and there was a significant negative correlation between WBC and HDL-C, which is consistent with the findings in the physical examination population [5–8], the population with impaired glucose regulation [9], type 2 diabetes mellitus (T2DM) population [10], and elderly population [11]. In addition, the BMI of the WBC ≤ 4 × 10^9^/L group was lower than that of the WBC > 4 × 10^9^/L group, and there was a positive correlation between WBC and BMI, which is generally consistent with the results of previous studies [12]. A correlation analysis of clinical data from 11526 medical examiners found that WBC was positively correlated with BMI after controlling for age [9]. It has been reported that there was a correlation between BMI and WBC in 215 elderly patients with hypertension [13].

Peripheral blood lymphocytes are an important component of cellular immunity, and their decrease often indicates that the body is in an immunosuppressed state, which may cause disease progression and complications [14]. In this study, the TG levels in the LYM#≤1.10 × 10^9^/L group were lower than that in the LYM#>1.10 × 10^9^/L group of adult patients with hyperglycemia, and a markedly positive correlation was exhibited between TG levels and LYM#. In addition, TG levels in adult patients with hyperglycemia had an effect on LYM#. Lymphopenia occurs in a variety of conditions, such as systemic lupus erythematosus (SLE) [15]. However, the causes of lymphopenia in patients with SLE are not fully clarified, and the possible mechanisms include the increase in lymphocyte destruction or apoptosis increased destruction or apoptosis of lymphocytes mediated by anti-lymphocyte antibodies, anti-Ro52 antibodies, abnormalities in the complement system associated with increased lymphocyte apoptosis, changes in lymphocyte subpopulations prompted by decreased IL-2 levels contributing to lymphopenia in SLE, and the application of infections, hormones, and immunosuppressive agents can suppress immunoreactive cells, especially lymphocytes [16]. Currently, there are fewer reports concerning the factors affecting lymphocytes in people with hyperglycemia, and it deserves further study. We also found that the serum P levels were lower in the LYM#≤1.10 × 10^9^/L group and positively correlated with LYM#, and the serum P levels were the risk factor of LYM#≤1.10 × 10^9^/L. Our findings are consistent with previous studies in patients with bloodstream infections [17]. It was proved that age, the existence of tumors, and hypophosphatemia were independent influencing factors of lymphocyte reduction in 204 patients with bloodstream infections [17]. The cause of decreased lymphocytes may be related to lymphocyte apoptosis, and hypophosphatemia accompanied by lymphocyte reduction, suggesting that hypophosphatemia may be associated with abnormal immune function [17].

Eosinophilia is one of the common features of hypersensitivity reactions caused by the sequential release of antigen-activated Th2 cells and key cytokines, including the release of key cytokines such as IL-5, which trigger eosinophil proliferation, accumulation, and function. Hypersensitivity reactions are one of the causes of eosinophilia in patients with chronic kidney disease (CKD). Medications, infections, and autoimmune diseases may also contribute to eosinophilia in patients with acute interstitial nephritis. The higher the CKD stage in patients with heart disease, the higher the prevalence of eosinophilia, which may be associated with subclinical cholesterol embolism, drug allergy, and contrast nephropathy [18]. In the present study, our findings indicated that UA levels in the EO#>0.14 × 10^9^/L group were higher than in the EO#≤0.14 × 10^9^/L group of patients with hyperglycemia, and UA was significantly positively correlated with EO#. Moreover, our regression analysis revealed that UA levels were a risk factor of EO#, which was slightly different from the previous research in patients with CKD. Huanying et al. found that eosinophil counts were negatively correlated with estimated glomerular filtration rate and positively correlated with urea, Cre, cystatin C, and retinol-binding protein, and eosinophil counts were positively correlated with UA but were not yet statistically significant and needs to be analyzed a larger sample size [18]. At present, the relationship between blood AU and EO# in patients with hyperglycemia is less reported and requires further investigation.

In the study, the age of the RDW − CV > 12.90 g/L group was significantly higher than that of the RDW − CV ≤ 12.90 g/L group, and there was a positive correlation between age and RDW-CV. Currently, most studies on the effect of age on RDW-CV have focused on coronary heart disease (CHD) [19, 20], hypertension [21], cerebral infarction [22], and hemodialysis [23] populations, with fewer studies related to hyperglycemic populations. A retrospective study found that age was an independent and positively correlated risk factor affecting the REW-CV of patients with peritoneal dialysis [24]. It has also been demonstrated in another retrospective study of esophageal cancer patients that the increased risk of RDW-CV was 2.989 times higher in patients over 60 years of age than in patients under 60 years of age, and age was positively correlated with RDW-CV [25]. The results of the present study are consistent with these studies, both suggesting a positive correlation between age and RDW-CV. Moreover, FBG was decreased in the RDW − CV > 12.90 g/L group than in the RDW − CV ≤ 12.90 g/L group and was significantly and negatively correlated with RDW-CV levels. It has been proved that RDW-CV was positively correlated with FBG in over 200 patients with T2DM [26, 27]. It has also been shown that RDW was negatively correlated with FBG in 256 patients with cerebral infarction [28]. Besides, it was found that there was no correlation between RDW and FBG in patients with metabolic syndrome [29]. Since the research on the correlation between RDW-CV and FBG is less, the results are not yet consistent, and the correlation is not yet clear, so the sample size needs to be expanded for further studies.

At present, there have been few studies and inconsistent results regarding the effect of HDL-C on Fib levels. Regression analysis of studies on patients with CHD has found a negative correlation between Fib levels and HDL-C [30]. In addition, it was found that there is a negative correlation between HDL-C and plasma Fib levels by comparing the physical examination of people with Fib > 4 g/L and those with Fib < 4 g/L [31]. The results of the present study showed that HDL-C levels in the group with Fib > 4 g/L were lower than HDL-C levels in the group with fibrinogen ≤ 4 g/L and were significantly negatively correlated with Fib levels, and the results of the present study were consistent with the above studies. However, it has been reported that in the clinical data of inpatients with a primary diagnosis of T2DM, regression analysis of the relationship between plasma Fib and age, disease duration, and metabolic indicators of patients showed that other independent variables such as HDL-C did not enter the regression model, which is different from the results of the present study [32]. It may be related to the selection of the study group and the sample size, and the sample size can be expanded to analyze subgroups of the population with different degrees of elevated blood glucose in further study.

In this study, we found that the TG level in the NSE ≤ 16.3 ng/mL group was lower than that in the NSE > 16.3 ng/mL group of adult patients with hyperglycemia, and there was a positive correlation between TG and NSE levels. It has been reported that increased TG was positively associated with NSE levels in patients with ischemic stroke with NSE hyperplasia (NSE > 13 *μ*g/mL), and TG and NSE levels were positively correlated in elderly lung cancer patients without surgery and chemotherapy [33, 34], which is similar to the results of the present study. Currently, the correlation between blood TG levels and blood NSE levels is poorly reported and has not been reported in hyperglycemic populations. The mechanism of the effect of TG on NSE levels is unclear and needs to be further investigated.

In the present study, Mg levels in the CEA > 5.0 ng/mL group were lower and negatively correlated with CEA levels, while FBG levels in the CEA > 5.0 ng/mL group were lower and positively correlated with CEA levels, compared with the CEA ≤ 5.0 ng/mL group. There are findings showing a significant positive correlation between CEA and FBG in the T2DM population and nonalcoholic steatosis patients [35–37], which is consistent with the results of the present study. However, there was a study reported that no correlation was found between the FBG and CEA levels in a population study of 150 T2DM patients; it may be limited by the small sample size of that study, which needs to be further analyzed after expanding the sample size [38]. In addition, as no literature reports on the correlation between Mg and CEA levels have been retrieved, the mechanism of the effect of Mg on CEA levels is unclear and needs to be further investigated.

Our findings demonstrated that compared with CA50 ≤ 7.20 U/mL, UA was lower and FBG was higher in the CA50 > 7.20 U/mL group; there was a significant negative correlation between UA and CA50 levels and a positive correlation between FBG and CA50 levels. It has not been reported on the correlation between CA50 and UA, and the mechanism by which UA affects CA50 levels is unclear and needs to be further investigated. Nevertheless, there is less literature on the correlation between CA50 and FBG; it was reported that high and low levels of CA50 in patients with T2DM were not associated with FBG levels [39], which is inconsistent with our findings. Notably, only 76 patients with T2DM were included in the above study; the results may be related to the small sample size; HbA1c is closely associated with FBG, which needs to be further verified by expanding the sample size.

It has been reported that Mg levels were significantly negatively correlated with FBG in T2DM patients [40, 41] and acute cerebrovascular patients [42]. There is a positive correlation between K and Mg levels in the patients with nonketotic DM combined with hypokalemia [42], and a positive correlation between Zn and Mg was observed in the physical examination population of 2122 children [43]. It has been found that the Mg level was positively correlated with C-P in 196 gestational diabetes patients [31]. Our findings suggested that FBG was significantly negatively correlated with Mg levels, and C-P, K, and Zn levels were significantly positively correlated with Mg levels, which were consistent with the previous studies. However, there are fewer studies on the relationship between Mg and C-P, and the conclusions are inconsistent. Jinquan et al. found that Mg level was not correlated with C-P in the 80 patients in the intensive care unit, but the sample size of this study was small and further studies with larger sample sizes are required for validation [44].

However, the study has some limitations. This is a single-center retrospective study with limited sample size, and due to incomplete or limited exclusion criteria, it is possible that patients with other comorbidities [45] that may affect blood glucose and patients who may be using medications that may cause hyperglycemia or changes in examination levels [46, 47] were included in this study. Moreover, insulin-like growth factor- (IGF-) 1 and adipokines like leptin have been known to be involved in the context of obesity. Investigations of the two factors in this study were absent; thus, further studies require to link them with other factors in hyperglycaemia from the perspective of pathophysiology.

## 5. Conclusions

In summary, endocrine metabolic factors are closely related to hemocyte parameters, tumor markers, and blood electrolytes in patients with hyperglycemia. Our findings provide great referential values for further clinical studies.

## Figures and Tables

**Table 1:** Comparison of clinical data of patients in the WBC ≤ 4 × 10^9^/L and WBC > 44 × 10^9^/L groups.

Items	WBC ≤ 4 × 10^9^/L (*n* = 90)	WBC > 4 × 10^9^/L (*n* = 1701)	*P* value
Male/female	43/47	915/786	0.265
Average age (years)	56	54	0.084
AST (U/L)	18.8	18.1	0.415
ALT (U/L)	18.3	19.5	0.244
Cre (*μ*mol/L)	64.9	70	0.014
UA (*μ*mol/L)	315.3	343.1	0.001
FBG (mmol/L)	5.75	6.1	0.467
TC (mmol/L)	4.065	4.22	0.209
F-CHOL	1.36	1.4	0.105
TG (mmol/L)	1.23	1.59	0.001
HDL-C (mmol/L)	1.11	1.02	0.001
LDL-C (mmol/L)	2.55	2.61	0.119
BMI (kg/m^2^)	22.6809	24.2215	0.000

Note: data are shown as mean (independent sample *t*-test). WBC: white blood cell count; AST: aspartate aminotransferase; ALT: alanine aminotransferase; Cre: creatinine; UA: uric acid; FBG: fasting blood glucose; TC: total cholesterol; F-CHOL: free cholesterol; TG, triglyceride; HLD-C: high-density lipoprotein cholesterol; LDL-C: low-density lipoprotein cholesterol; BMI: body mass index.

**Table 2:** Comparison of clinical data of patients in the LYM#≤1.10 × 10^9^/L and LYM#>1.10 × 10^9^/L groups.

Items	LYM#≤1.10 × 10^9^/L (*n* = 63)	LYM#>1.10 × 10^9^/L (*n* = 1541)	*Z* or *χ*2	*P* value
LYM# (×10^9^/L)	0.91	2.14	-13.471	0.001
Average age (years)	66	54	-5.092	0.001
AST (U/L)	18.3	18.0	-0.530	-0.596
ALT (U/L)	16.6	19.4	-1.695	0.090
Cre (*μ*mol/L)	70.1	70.0	-1.059	0.290
UA (*μ*mol/L)	313.8	339.5	-2.416	0.016
FBG (mmol/L)	6.2	6.1	-0.437	0.662
TC (mmol/L)	3.9	4.19	-2.991	0.003
F-CHOL	1.27	1.4	-2.373	0.018
TG (mmol/L)	1.18	1.57	-3.865	0.000
HDL-C (mmol/L)	0.99	1.02	-0.228	0.819
LDL-C (mmol/L)	2.37	2.61	-2.681	0.007
K (mmol/L)	3.63	3.58	-0.081	0.936
Na (mmol/L)	143.0	143.1	-0.750	0.453
Cl (mmol/L)	105.9	105.5	-0.081	0.935
Ca (mmol/L)	2.27	2.3	-2.250	0.024
Mg (mmol/L)	0.83	0.84	-0.776	0.437
P (mmol/L)	0.93	1.11	-7.001	0.001
Zn (mmol/L)	9.88	10.63	-2.410	0.016
BMI (kg/m^2^)	23.3844	24.2188	-2.230	0.026

Note: data are shown as mean (independent sample *t*-test). LYM#: absolute lymphocyte; AST: aspartate aminotransferase; ALT: alanine aminotransferase; Cre: creatinine; UA: uric acid; FBG: fasting blood glucose; TC: total cholesterol; F-CHOL: free cholesterol; TG: triglyceride; HLD-C: high-density lipoprotein cholesterol; LDL-C: low-density lipoprotein cholesterol; Ka: serum kalium; Na: serum sodium; Cl: serum chlorine; Ca: serum calcium; Mg: serum magnesium; P: serum phosphorus; Zn: serum zinc; BMI: body mass index.

**Table 3:** Comparison of clinical data of patients in the EO#≤0.14 × 10^9^/L and EO#>0.14 × 10^9^/L groups.

Items	EO% ≤ 0.14 × 10^9^/L (*n* = 924)	EO% > 0.14 × 10^9^/L (*n* = 842)	*P* value
Male/female	414/510	535/307	0.001
Average age (years)	54	53	0.516
AST (U/L)	17.8	18.3	0.114
ALT (U/L)	18.7	20.1	0.001
Cre (*μ*mol/L)	67.5	72.9	0.001
UA (*μ*mol/L)	330.45	359.0	0.001
FBG (mmol/L)	6.0	6.1	0.853
TC (mmol/L)	4.24	4.19	0.359
F-CHOL	1.41	1.4	0.882
TG (mmol/L)	1.56	1.64	0.007
HDL-C (mmol/L)	1.05	0.98	0.001
LDL-C (mmol/L)	2.62	2.60	0.524
BMI (kg/m^2^)	23.87	24.57	0.001

Note: data are shown as mean (independent sample *t*-test). EO#: absolute eosinophil value; AST: aspartate aminotransferase; ALT: alanine aminotransferase; Cre: creatinine; UA: uric acid; FBG: fasting blood glucose; TC: total cholesterol; F-CHOL: free cholesterol; TG: triglyceride; HLD-C: high-density lipoprotein cholesterol; LDL-C: low-density lipoprotein cholesterol; Ka: serum kalium; Na: serum sodium; Cl: serum chlorine; Ca: serum calcium; Mg: serum magnesium; P: serum phosphorus; Zn: serum zinc; BMI: body mass index.

**Table 4:** Comparison of clinical data of patients in the RDW − CV ≤ 12.90 g/L and RDW − CV > 12.90 g/L groups.

Items	RDW − CV ≤ 12.90 g/L (*n* = 903)	RDW − CV > 12.90 g/L (*n* = 862)	*Z* or *χ*2	*P* value
Male/female	519/384	426/436	11.504	0.001
Average age (years)	52	57	-7.622	0.001
AST (U/L)	18.1	18.0	-1.150	0.250
ALT (U/L)	20.10	18.95	-1.781	0.075
Cre (*μ*mol/L)	70.0	70.05	-1.950	0.051
UA (*μ*mol/L)	344.10	341.6	-0.745	0.456
FBG (mmol/L)	6.3	5.7	-4.934	0.001
TC (mmol/L)	4.18	4.25	-0.357	0.721
F-CHOL	1.42	1.39	-1.666	0.096
TG (mmol/L)	1.65	1.525	-2.702	0.007
HDL-C (mmol/L)	0.98	1.05	-3.898	0.001
LDL-C (mmol/L)	2.61	2.59	-1.123	0.261
K (mmol/L)	2.59	3.6	-1.149	0.251
Na (mmol/L)	142.8	143.4	-4.407	0.001
Cl (mmol/L)	105.0	105.8	-4.481	0.001
Ca (mmol/L)	2.31	2.30	-1.109	0.267
Mg (mmol/L)	0.83	0.84	-1.090	0.276
P (mmol/L)	1.12	1.09	-3.397	0.001
Zn (mmol/L)	10.67	10.57	-1.432	0.152
BMI (kg/m^2^)	24.2215	24.2215	-0.254	0.799

Note: data are shown as mean (independent sample *t*-test). RDW-CV: red blood cell distribution width-coefficient of variation; absolute lymphocyte; AST: aspartate aminotransferase; ALT: alanine aminotransferase; Cre: creatinine; UA: uric acid; FBG: fasting blood glucose; TC: total cholesterol; F-CHOL: free cholesterol; TG: triglyceride; HLD-C: high-density lipoprotein cholesterol; LDL-C: low-density lipoprotein cholesterol; Ka: serum kalium; Na: serum sodium; Cl: serum chlorine; Ca: serum calcium; Mg: serum magnesium; P: serum phosphorus; Zn: serum zinc; BMI: body mass index.

**Table 5:** Comparison of clinical data of patients in the Fib ≤ 4 g/L and Fib > 4 g/L groups.

Items	Fib ≤ 4 g/L (*n* = 1441)	Fib > 4 g/L (*n* = 169)	*Z* or *χ*2	*P* value
Male/female	766/675	92/77	0.1	0.752
Average age (years)	53	58	-3.043	0.002
AST (U/L)	18.3	16.4	-2.974	0.003
ALT (U/L)	20.0	17.1	-3.129	0.002
Cre (*μ*mol/L)	69.3	74.6	-3.946	0.001
UA (*μ*mol/L)	341.2	335.6	-0.749	0.454
FBG (mmol/L)	6.0	6.8	-3.559	0.001
TC (mmol/L)	4.21	4.23	-0.112	0.910
F-CHOL	1.39	1.44	-1.745	0.081
TG (mmol/L)	1.59	1.57	-0.243	0.808
HDL-C (mmol/L)	1.02	0.93	-3.868	0.001
LDL-C (mmol/L)	2.6	2.67	-0.786	0.432
K (mmol/L)	3.57	3.66	-4.057	0.001
Na (mmol/L)	143.2	142.0	-4.474	0.001
Cl (mmol/L)	105.4	104.8	-2.710	0.007
Ca (mmol/L)	2.3	2.32	-1.347	0.178
Mg (mmol/L)	0.83	0.84	-1.141	0.254
P (mmol/L)	1.12	1.06	-2.731	0.006
Zn (mmol/L)	10.78	10.19	-2.904	0.004
BMI (kg/m^2^)	24.141	24.56	-1.346	0.178

Note: data are shown as mean (independent sample *t*-test). Fib: fibrinogen; AST: aspartate aminotransferase; ALT: alanine aminotransferase; Cre: creatinine; UA: uric acid; FBG: fasting blood glucose; TC: total cholesterol; F-CHOL: free cholesterol; TG: triglyceride; HLD-C: high-density lipoprotein cholesterol; LDL-C: low-density lipoprotein cholesterol; Ka: serum kalium; Na: serum sodium; Cl: serum chlorine; Ca: serum calcium; Mg: serum magnesium; P: serum phosphorus; Zn: serum zinc; BMI: body mass index.

**Table 6:** Comparison of clinical data of patients in the NSE ≤ 16.3 ng/mL and NSE > 16.3 ng/mL groups.

Items	NSE ≤ 16.3 ng/mL (*n* = 753)	NSE > 16.3 ng/mL (*n* = 20)	*Z* or *χ*2	*P* value
Male/female	415/338	13/7	0.771	0.380
Average age (years)	54	46	-2.379	0.017
AST (U/L)	18.2	20.35	-0.887	0.375
ALT (U/L)	19.7	23.05	-0.615	0.538
Cre (*μ*mol/L)	70.6	59.6	-1.324	0.185
UA (*μ*mol/L)	347.4	383.4	-0.545	0.586
FBG (mmol/L)	6.0	7.425	-1.577	0.115
TC (mmol/L)	4.15	4.71	-2.281	0.023
F-CHOL	1.43	1.75	-3.270	0.001
TG (mmol/L)	1.60	2.335	-2.790	0.005
HDL-C (mmol/L)	1.02	0.95	-0.590	0.555
LDL-C (mmol/L)	2.54	3.005	-1.621	0.105
BMI (kg/m^2^)	24.141	23.90	-0.919	0.358

Note: data are shown as mean (independent sample *t*-test). NSE: neuron-specific enolase; AST: aspartate aminotransferase; ALT: alanine aminotransferase; Cre: creatinine; UA: uric acid; FBG: fasting blood glucose; TC: total cholesterol; F-CHOL: free cholesterol; TG: triglyceride; HLD-C: high-density lipoprotein cholesterol; LDL-C: low-density lipoprotein cholesterol; Ka: serum kalium; Na: serum sodium; Cl: serum chlorine; Ca: serum calcium; Mg: serum magnesium; P: serum phosphorus; Zn: serum zinc; BMI: body mass index.

**Table 7:** Comparison of clinical data of patients in the CEA ≤ 5.0 ng/mL and CEA > 5.0 ng/mL groups.

Items	CEA ≤ 5.0 ng/mL (*n* = 668)	CEA > 5.0 ng/mL (*n* = 102)	*Z* or *χ*2	*P* value
Male/female	356/312	69/33	7.372	0.007
CEA	2.37	6.47	-16.283	0.001
Average age (years)	53	55	-1.569	0.117
AST (U/L)	18.2	18.9	-1.343	0.179
ALT (U/L)	19.65	20.2	-0.290	0.772
Cre (*μ*mol/L)	70.3	70.8	-1.102	0.270
UA (*μ*mol/L)	344.85	359.05	-0.602	0.547
FBG (mmol/L)	5.88	7.35	-3.770	0.001
TC (mmol/L)	4.15	4.43	-1.377	0.168
F-CHOL	1.43	1.52	-2.055	0.040
TG (mmol/L)	1.62	1.535	-0.931	0.352
HDL-C (mmol/L)	1.015	1.04	-1.515	0.130
LDL-C (mmol/L)	2.53	2.66	-0.810	0.418
K (mmol/L)	3.56	3.495	-1.126	0.260
Na (mmol/L)	142.9	142.6	-1.438	0.151
Cl (mmol/L)	105.7	105.2	-1.492	0.136
Ca (mmol/L)	2.3	2.3	-0.601	0.548
Mg (mmol/L)	0.84	0.815	-3.188	0.001
P (mmol/L)	1.12	1.08	-0.722	0.470
Zn (mmol/L)	10.22	9.635	-1.641	0.101
BMI (kg/m^2^)	24.195	23.91	-1.942	0.052

Note: data are shown as mean (independent sample *t*-test). CEA: carcinoembryonic antigen; AST: aspartate aminotransferase; ALT: alanine aminotransferase; Cre: creatinine; UA: uric acid; FBG: fasting blood glucose; TC: total cholesterol; F-CHOL: free cholesterol; TG: triglyceride; HLD-C: high-density lipoprotein cholesterol; LDL-C: low-density lipoprotein cholesterol; Ka: serum kalium; Na: serum sodium; Cl: serum chlorine; Ca: serum calcium; Mg: serum magnesium; P: serum phosphorus; Zn: serum zinc; BMI: body mass index.

**Table 8:** Comparison of clinical data of patients in the CA50 ≤ 7.20 U/mL and CA50 > 7.20 U/mL groups.

Items	CA50 ≤ 7.20 U/mL (*n* = 324)	CA50 > 7.20 U/mL (*n* = 324)	*Z* or *χ*2	*P* value
CA50	3.865	12.795	-22.028	0.001
Average age (years)	52	55	-4.212	0.001
AST (U/L)	18.2	18.3	-0.689	0.491
ALT (U/L)	20.1	19.55	-0.438	0.661
Cre (*μ*mol/L)	69.95	70.3	-0.095	0.924
UA (*μ*mol/L)	365.9	334.75	-2.946	0.003
FBG (mmol/L)	5.4	6.4	-5.654	0.001
TC (mmol/L)	4.14	4.23	-0.983	0.326
F-CHOL	1.41	1.48	-1.811	0.070
TG (mmol/L)	1.56	1.665	-1.269	0.204
HDL-C (mmol/L)	1.01	1.04	-0.620	0.535
LDL-C (mmol/L)	2.565	2.585	-0.217	0.829
K (mmol/L)	3.565	3.575	-0.320	0.749
Na (mmol/L)	143.3	142.9	-1.599	0.110
Cl (mmol/L)	105.8	105.6	-0.910	0.363
Ca (mmol/L)	2.3	2.31	-0.257	0.798
Mg (mmol/L)	0.84	0.84	-1.216	0.224
P (mmol/L)	1.15	1.11	-2.280	0.023
Zn (mmol/L)	10.215	9.935	-1.646	0.100
BMI (kg/m^2^)	24.0346	24.3375	-0.037	0.971

Note: data are shown as mean (independent sample *t*-test). CA50: tumor marker; AST: aspartate aminotransferase; ALT: alanine aminotransferase; Cre: creatinine; UA: uric acid; FBG: fasting blood glucose; TC: total cholesterol; F-CHOL: free cholesterol; TG: triglyceride; HLD-C: high-density lipoprotein cholesterol; LDL-C: low-density lipoprotein cholesterol; Ka: serum kalium; Na: serum sodium; Cl: serum chlorine; Ca: serum calcium; Mg: serum magnesium; P: serum phosphorus; Zn: serum zinc; BMI: body mass index.

**Table 9:** Comparison of clinical data of patients in the Mg ≤ 0.75 mmol/L and Mg > 0.75 mmol/L groups.

Items	Mg ≤ 0.75 mmol/L (*n* = 141)	Mg > 0.75 mmol/L (*n* = 854)	*Z* or *χ*2	*P* value
C-P (ng/mL)	0.91	0.14	-2.613	0.009
Male/female	81/60	455/399	0.846	0.358
Average age (years)	52	54	-1.762	0.078
AST (U/L)	17.3	18.2	-0.652	0.514
ALT (U/L)	19.5	20.0	-0.465	0.642
Cre (*μ*mol/L)	68.9	70.0	-0.717	0.474
UA (*μ*mol/L)	351.1	348.15	-0.367	0.713
FBG (mmol/L)	7.4	5.9	-4.482	0.001
TC (mmol/L)	3.97	4.23	-2.449	0.014
F-CHOL	1.42	1.48	-1.580	0.114
TG vmmol/L)	1.67	1.62	-0.246	0.806
HDL-C (mmol/L)	0.98	1.01	-1.099	0.272
LDL-C (mmol/L)	2.26	2.595	-2.807	0.005
K (mmol/L)	3.43	3.6	-4.140	0.001
Na (mmol/L)	142.3	143.3	-3.358	0.001
Cl (mmol/L)	105.7	105.9	-2.737	0.006
Ca (mmol/L)	2.29	2.3	-1.832	0.067
Mg (mmol/L)	0.71	0.86	-19.058	0.001
P (mmol/L)	1.1	1.12	-0.462	0.644
Zn (mmol/L)	9.64	10.14	-3.125	0.002
BMI (kg/m^2^)	24.4352	24.3496	-0.138	0.890

Note: data are shown as mean (independent sample *t*-test). C-P: fasting C-peptide; AST: aspartate aminotransferase; ALT: alanine aminotransferase; Cre: creatinine; UA: uric acid; FBG: fasting blood glucose; TC: total cholesterol; F-CHOL: free cholesterol; TG: triglyceride; HLD-C: high-density lipoprotein cholesterol; LDL-C: low-density lipoprotein cholesterol; Ka: serum kalium; Na: serum sodium; Cl: serum chlorine; Ca: serum calcium; Mg: serum magnesium; P: serum phosphorus; Zn: serum zinc; BMI: body mass index.

## Data Availability

The data that support the findings of this study are available from the corresponding author upon reasonable request.
